# Refugee and immigrant-background parents’ academic engagement, resources, and children’s adjustment in German lower-income elementary schools

**DOI:** 10.3389/fpsyg.2025.1550107

**Published:** 2025-09-10

**Authors:** S. Chwastek, B. Leyendecker, P. Jugert, N. McElvany, J. Busch

**Affiliations:** ^1^Child and Family Research, Faculty of Psychology, Ruhr-University, Bochum, Germany; ^2^Department of Psychology, University Duisburg-Essen, Essen, Germany; ^3^Center for Research on Education and School Development (IFS), TU Dortmund University, Dortmund, Germany

**Keywords:** refugee, immigrant, parental engagement, parental involvement, primary education, integration, poverty, quantitative

## Abstract

**Background:**

Refugee and recently immigrated (RRI) families in Germany often reside in lower-income, multiethnic neighborhoods alongside many other immigrant-background families. Their children are likely to face barriers to their academic development. Yet, research on how these parents’ academic engagement and resources influence their children’s school adjustment remains scarce. We explored (a) the relations of parents’ academic engagement, resources, and their children’s school adjustment, and (b) the exact forms of parents’ academic engagement and resources, facilitators, and barriers to it.

**Methods:**

We conducted structured telephone-based interviews including closed- and open-format questions with Arabic-speaking refugee (*refugee*), recently immigrated, immigrant-background, and non-immigrant parents in German lower-income neighborhoods (*N* = 108). Measures assessed relations between parents’ home and school engagement, parent- and school-related resources (formal education, mental health, German language skills, social support, educational aspirations, knowledge of the German education system, parent-teacher relationship, sense of well-being at school), and children’s elementary school adjustment (socio-emotional adjustment, grades, well-being at school).

**Results:**

Multiple regression analyses revealed that German language skills and participation in local social support groups were related to their home and school engagement. Parents’ academic engagement was not related to children’s school adjustment. RRI parents reported lower levels of resources than immigrant-background and non-immigrant parents, with refugee parents reporting the lowest levels of resources. Descriptive analyses of the open-format questions and single-scale items revealed group differences in parents’ school engagement, home engagement, and resources (e.g., perceived responsibilities, communication with school and other parents). Regarding other dimensions, RRI, immigrant-background, and non-immigrant parents reported similar levels of resources. Additionally, we found indicators of RRI parents’ high academic expectations and willingness to support their children academically.

**Conclusion:**

Our findings suggest the importance of increasing RRI and immigrant-background parents’ availability of resources to facilitate their academic engagement. Our findings suggest that their children may adjust to school independently of parents’ academic engagement as measured. Future research should examine the contribution of unmeasured (e.g., better culturally adapted) academic engagement forms as well as which school- and community-level factors may compensate for limited parental resources.

## 1 Introduction

For decades, Germany has been a country of immigration for people from diverse countries. In the German state of North Rhine-Westphalia (NRW), many lower-income, multiethnic neighborhoods were established. These neighborhoods now experience high population turnover. Many refugee, recently immigrated, and economically less stable families are likely to settle in these neighborhoods, while those who become more economically stable tend to move away. In previous decades, most immigrant families in Germany came from European countries, Turkey, and the former Soviet Union (BPB, 2018). During the past two decades, however, countries of origin have become increasingly diverse. Consequently, elementary schools in these neighborhoods have been struggling to prepare children for secondary school and to foster their development. Since the mid-2010s, a high number of Arabic-speaking refugee families has been moving into the already diverse neighborhoods (MKFFI, 2019). As a result, schools’ diversity regarding children’s cultures, languages, and (pre-) school experiences has been increasing considerably. By 2018, 43.2% of all children under the age of 18 in NRW were immigrant-background children (MAGS, 2020). In this study, we explored (a) the relations between parents’ academic engagement, their parent- and school-related resources, and their children’s school adjustment; as well as (b) the extent of the parents’ academic engagement and resources of three large groups of immigrant-background families in above-mentioned neighborhoods: Arabic-speaking refugee (*refugee*) families, recently immigrated families, and immigrant-background families.

### 1.1 Challenges to RRI families

Refugee families are a very specific group of (recently) immigrated families. According to UNHCR (2025), refugee families have been forced to flee their countries of origin, have sought safety in another country, and are likely not able to return to their countries of origin anytime soon. In contrast, most recently immigrated parents have likely moved to another country without force or threat. In our study, recently immigrated parents moved to Germany as adults, whereas immigrant-background parents have grown up in Germany and at least one parent was a second-generation immigrant. They have longer settlement histories compared to RRI families. Still, immigrant-background children show a lower school adjustment than their non-immigrant peers (e.g., Aghajafari et al., 2020; Wendt et al., 2020). Research on immigrant-background—especially refugee and recently immigrated (RRI) − families in the above-mentioned neighborhoods remains scarce. Knowledge of this study may contribute to understanding how parents’ resources, academic engagement, and children’s school adjustment interact in increasingly diverse, lower-income educational contexts. It may further shed light on RRI and immigrant-background parents’ resources in the above-mentioned neighborhoods. To do so, we conducted interviews with Arabic-speaking refugee (*refugee*), recently immigrated, immigrant-background, and non-immigrant parents living in the above-mentioned neighborhoods.

Living in lower-income neighborhoods is likely to be associated with fewer available resources (Barglowski, 2019). Especially RRI families share similar challenges in adapting to new educational systems and face comparable resource constraints, as they are in the early stages of the settlement process. For example, they may struggle with the unfamiliar German bureaucracy, language, and education system (González-Falcón et al., 2022). In addition, they lack first-hand experiences with the German education system and therefore feel less confident about how to support their children’s education, making it more challenging to meet the expectations of teachers (Cureton, 2020; Zengin and Akdemir, 2020). Although RRI parents may share many similar experiences, particularly refugee parents may have access to even fewer resources due to flight-related factors, such as traumatic events or uncertain residency status (Bronstein and Montgomery, 2011). While it is important to consider the potential differences between refugee and recently immigrated parents, it is equally essential to address the overall adjustment of RRI parents.

Despite adjustment difficulties, international studies found that RRI parents had high educational aspirations for their children (Stevenson and Willott, 2007) and were willing to support them (Baird, 2015; Gandarilla Ocampo et al., 2021). RRI and immigrant-background parents’ academic engagement was indeed found to foster their children’s school adjustment (Jeynes, 2003; Wong and Schweitzer, 2017). A lack of resources, however, was repeatedly found to impede parents’ academic engagement (e.g., Vera et al., 2012; Ziaian et al., 2018). Research on RRI parents in German lower-income neighborhoods is still scarce, as these parents are likely to move frequently, hesitate to participate in studies, and are less familiar with interviews and questionnaires. Additionally, little research has yet considered that different pre-, peri-, and post-migration experiences of refugee, recently immigrated, and immigrant-background families may affect parents’ academic engagement and their children’s school adjustment differently.

### 1.2 RRI and immigrant-background parents’ academic engagement

The Integrative Risk and Resilience Model by Suárez-Orozco et al. (2018) states that RRI and immigrant-background children’s school adjustment is influenced by several risk and protective factors. Several of these may lie within the contexts of the family, school, and neighborhood. In this study, we identify dimensions of parents’ academic engagement (i.e., home and school engagement) as possible risk and protective factors. Based on our literature review (in the following) and following Epstein’s (1987) well-established concepts, we define the dimensions of parents’ home engagement (i.e., cognitively stimulating activities with the child and homework-related activities) and parents’ school engagement (i.e., activities involving school, teachers, or other parents). These dimensions represent the most common forms of academic involvement observed in German elementary schools, as identified by Schwanenberg (2015) through her definitions of “learning-related involvement” (equivalent to home engagement) and “organizational involvement” (equivalent to school engagement).

To our knowledge, most authors (see Lavenda, 2011 for an exception) have found RRI parents to be less engaged at home and in school than non-immigrant parents (Baird, 2015; Baker et al., 2021; Cureton, 2020; Koyama and Bakuza, 2017; Zengin and Akdemir, 2020). However, several qualitative studies have identified home engagement strategies of RRI parents that prepared their children for school-related tasks (e.g., structuring time, staying informed, preparing material, or encouraging the child to seek help) or put the parents in contact with other parents and teachers at school (Cureton, 2020; Gandarilla Ocampo et al., 2021; Schlaich, 2021). In qualitative studies, refugee parents also reported supervising or controlling their children’s homework in a way that does not require content knowledge (Cureton, 2020; Shamim et al., 2020). Overall, these studies suggest that RRI parents are engaged in their children’s education, but either to a lesser extent or less visible to schools when compared to immigrant-background and non-immigrant parents. Less apparent homework engagement strategies were also reported by immigrant-background parents in some quantitative studies (e.g., Betz, 2005). Moreover, evidence suggests that parents’ academic engagement strategies may differ between ethnic groups (García Coll et al., 2002). For example, some authors also found that specific ethnic groups may prioritize home engagement over school engagement (Lin and Yang, 2024; Vera et al., 2012).

Taken together, the literature suggests that RRI parents highly value their children’s education, yet their supporting strategies may differ from those commonly expected in schools in higher-income countries. Prior research also suggests that RRI and immigrant-background parents’ academic engagement may differ within (e.g., cultural) subgroups. Up to this date, most studies on RRI parents’ academic engagement appear to be of a qualitative nature (Molla, 2024). The lack of rigorous quantitative research encompassing RRI parents’ academic engagement limits the generalizability of evidence. To develop comprehensive quantitative approaches, we also need qualitative insights into the academic engagement strategies employed by RRI and immigrant-background parents that remain invisible within the school context. Our study intends to combine these two approaches to shed light on RRI parents’ academic engagement and resources. It aims to apply quantitative instruments adapted to RRI parents’ academic engagement strategies but also uses open-format questions.

### 1.3 RRI and immigrant-background parents’ resources

We consider two dimensions of RRI and immigrant-background parents’ resources: Parent-related resources, which pertain to parents’ attributes or are directly shaped by them (e.g., formal education), and school-related resources, which pertain to parents’ feelings toward, knowledge of, or interactions with school (e.g., parent-teacher relationship). To date, mainly qualitative studies suggest that these resources could influence RRI parents’ academic engagement strategies (Gandarilla Ocampo et al., 2021; Schlaich, 2021) and that RRI parents are more likely to either lack certain resources or to prioritize different aspects of academic engagement. However, quantitative evidence on these relations, especially within samples of RRI parents, remains scarce. RRI and immigrant-background parents may have lower parent-related resources such as lower formal education, higher levels of distress (including non-flexible work schedules and lower income), and limited social support (González-Falcón et al., 2022; Kuhnt, 2017) when compared to non-immigrant families. Additionally, literature suggests that RRI parents may have even fewer resources than immigrant-background parents. Those may include their formal education (Ziaian et al., 2018), their language barriers and unfamiliarity with communication patterns in the receiving society (Cranston et al., 2021; Rah et al., 2009), immigration-related distress and mental health problems (Baker et al., 2021; Ziaian et al., 2018), social support (Young, 2001; Ziaian et al., 2018), and time constraints (Gandarilla Ocampo et al., 2021; Rah et al., 2009; Shamim et al., 2020). However, few studies have linked the extent of these resources to RRI parents’ home and school engagement (e.g., Baker et al., 2021; Rah et al., 2009). Adding to the body of literature at hand, we aim to explore the extent of RRI and immigrant-background parents’ parent-related resources as well as whether the extent of these resources is related to their levels of academic engagement at home and in school.

Several school-related resources of RRI and immigrant-background parents are likely to influence their home and school engagement. Such school-related resources may be their knowledge of the receiving country’s education system (Gandarilla Ocampo et al., 2021; Vera et al., 2012), their relationship with the teacher (Grace and Gerdes, 2019; Nzinga-Johnson et al., 2009), and their well-being at school (Cureton, 2020; Vera et al., 2012). For example, several authors found that RRI and immigrant-background parents perceived themselves as responsible for their children’s behavior and homework, but saw the responsibility for their children’s learning with the teachers (Cureton, 2020; Rah et al., 2009; Snell, 2018; Vera et al., 2012). This may result in more home engagement, but less school engagement. Insecurity about their role in their children’s education (Koyama and Bakuza, 2017; Sainsbury and Renzaho, 2011) or lesson content (Barglowski, 2019; Cureton, 2020) may additionally hamper RRI and immigrant-background parents’ academic engagement. Moreover, when lacking some of these parent- or school-related resources, parents may find it more difficult to communicate with the school staff or participate in school-related activities. We thus aim to explore the extent of RRI and immigrant-background parents’ school-related resources as well as whether the extent of these resources is related to their levels of academic engagement at home and in school.

We consider school adjustment as children’s successful adaptation to the school environment, encompassing both socio-emotional aspects and academic performance. RRI and immigrant-background parents’ academic engagement has repeatedly been linked to their children’s school adjustment (Areepattamannil and Lee, 2014; Jeynes, 2003; McBrien, 2005). Importantly, different dimensions of academic engagement may affect specific aspects of school adjustment in distinct ways. However, the exact nature of these relationships remains unclear. For example, some studies only associated parents’ home engagement, but not school engagement, with their children’s academic achievement (Altschul, 2011). Others linked engagement only to certain socio-emotional behaviors (Motti-Stefanidi et al., 2015) or associated certain aspects of engagement (e.g., certain homework rules or parenting styles) even negatively with children’s school adjustment (Jeynes, 2003).

### 1.4 This study

Previous work suggests that RRI and immigrant-background parents’ resources may influence their academic engagement, which, in turn, may relate to their children’s school adjustment. Still, evidence on which resources exactly may influence the academic engagement of immigrant-background parents, especially RRI parents, and whether parent- and school-related resources differ between refugee, recently immigrated, and immigrant-background parents remains scarce. Only very few quantitative studies on RRI parents’ academic engagement exist (Molla, 2024). Additionally, little research has yet linked distinct pre-, peri-, and post-migration experiences of RRI and immigrant-background families to parents’ academic engagement and their children’s school adjustment. This exploratory study aims to refine our understanding of (a) the relations of RRI and immigrant-background parents’ academic engagement, their resources, and their children’s school adjustment; as well as (b) the extent of RRI and immigrant-background parents’ academic engagement and resources.

*RQ1*: Do RRI and immigrant-background parents’ resources relate to their home and school engagement?

*RQ2*: Do their home and school engagement relate to their children’s school adjustment?

*RQ3*: Do parents’ academic engagement, resources, and their children’s school adjustment differ between (a) refugee, (b) recently immigrated, (c) immigrant-background, and (d) non-immigrant families within the same lower-income neighborhoods?

*RQ4*: What are the exact forms and strategies of RRI parents’ academic engagement, and which are influencing barriers and facilitators to it?

## 2 Materials and methods

### 2.1 Participants and procedure

We conducted telephone-based structured interviews in Arabic, German, or Turkish with *N_total_* = 108 parents (*M* = 39.33 years, *SD* = 5.99; 85.19% female) between November 2021 and August 2022 in the Ruhr Area in Germany. All participants were parents of a third- or fourth-grader in multiethnic, lower-income neighborhoods. Parents were primarily recruited through schools via leaflets in the respective languages (Arabic, German, Turkish) as part of a broader research project (*n* = 84). Research assistants recruited *n* = 24 additional parents within the same neighborhoods by approaching them at mosques, mosque associations, sports clubs, and via social media networking (*n* = 24). Information about the study and inclusion criteria was delivered orally and via leaflets. To ensure the comparability of parents’ living circumstances, we set the following eligibility criteria: (a) either the interviewee or both of their parents were born abroad, and the interviewee did not complete the highest German school leaving certificate (*recently immigrated* and *immigrant-background* groups) or (b) the interviewee was an Arabic-speaking refugee (*refugee* group). The parents sent us their contact data via letter mail or e-mail, either via the school or by themselves. At the beginning of the telephone-based interview, the research assistants verified that all participants met the inclusion criteria. Of initially *N* = 200 interested parents, *n* = 108 agreed to the interview (*n* = 92 parents declined or did not answer our messages and telephone calls). The parents received further study information via letter mail and returned a signed informed consent form. They received 20€ for participation. Research assistants who were native speakers were trained to conduct the structured interviews and provide adequate information to the parents without being suggestive. We conducted structured interviews to ensure that participants understood all items. These structured interviews encompassed both closed- and open-format questions.

We divided the parents into four groups for group comparisons (RQ3 and RQ4). The *refugee* group consisted of *n =* 23 Arabic-speaking refugee parents (age: *M* = 36.17, *SD* = 5.42; 74% female). The *recently immigrated* group consisted of *n =* 22 parents (age: *M* = 42.27, *SD* = 5.06; 82% female). The *immigrant-background* group consisted of *n =* 28 families with one or both parents born in Germany, but at least one parent was a second-generation immigrant (age: *M* = 37.39, *SD* = 5.29; 96% female). Prior, we excluded *n* = 6 parents from the *recently immigrated* group who immigrated to Germany before reaching the age of 17. We also excluded *n* = 7 parents from the *immigrant-background* group who immigrated to Germany after reaching the age of 17. Thus, we ensured that only recently immigrated parents were in the recently immigrated group as well as that none of the recently immigrated parents were within the immigrant-background group. We differed between recently immigrated and immigrant-background parents, because we presumed that parents who were not enrolled in the German school system might have more difficulties in meeting the school’s expectations of the parents’ role in their children’s education and thus might be engaged in a different manner than parents who were enrolled in the German school system. The *non-immigrant* group consisted of *n =* 22 non-immigrant parents (age: *M* = 41.91, *SD* = 6.64; 86% female). The internal review board of the Ethics Committee of the Faculty of Psychology of the Ruhr-University Bochum approved the study protocol. General Data Protection Regulation requirements were met. Tables 1–3 display demographic variables of the parents and their children within the different groups.

**Table 1:** Descriptives on parents − part one.

	*M*	*SD*	*Mdn*	*Min*	*Max*
Time in Germany in years					
*Refugee* (*n* = 23)	6.35	3.74	6	2	18
*Recently arrived* (*n* = 22)	13.45	7.76	12	0	25
Child—age in months					
(*refugee*)	121.26	14.28	126	94	144
(*recently*)	124.29	11.74	124.5	106	168
(*immig*)	120.93	17.86	119	96	207
(*non-imm.*)	123.45	6.70	122	112	135
Household: persons/rooms-ratio					
(*refugee*)	1.54	0.38	1.60	1.00	2.33
(*recently*)	1.32	0.52	1.25	0.60	3.00
(*immig*)	1.25	0.45	1.25	0.57	2.50
(*non-imm.*)	0.96	0.24	1.00	0.50	1.33

*n* = number of cases. *M* = mean. *SD* = standard deviation. *Mdn* = median. *Min* = minimum. *Max* = maximum. If *n* is not indicated, all parents of the group replied to the question. Refugee = both parents refugee parents. Recently = recently immigrated parents. Immig. = Immigrant-background (either one of the parents born abroad or at least one of the parents having parents which were born abroad). Non-immig. = Non-immigrant parents.

**Table 2:** Descriptives on parents − part two.

Descriptives		Refugee^a^	Recently^a^	Immig.^a^	Non-imm.^a^
Living with partner	Yes	86.96	63.64	92.86	72.73
Number of children	1	–	–	3.57	13.64
	2–3	60.87	68.18	71.43	77.27
	4–6	39.13	31.82	25.00	9.09
Country of birth	Germany	–	–	78.57	100.00
	Arab countries^b^	100.00	22.73	3.57	–
	Europe, East^c^	–	36.36	17.85	–
	Other^d^	–	40.90	–	–
Partner country of birth (*n* = 22; 14, 33, 16)	Germany	–	–	57.69	100.00
	Arab countries	100.00	20.00	11.54	–
	Europe, East	–	53.34	26.93	–
	Other	–	26.67	3.85	–
Residency status (*n* = 23; 21; 19; 22)	Permanent	13.04	57.14	73.68	100.00
At least one parent currently working	Yes	65.22	63.64	85.71	86.36
Language spoken at home	German	4.35	9.09	21.43	95.24
	Arabic	65.25	–	–	–
	German and another language	8.70	45.44	53.57	4.76
	Other	21.74	45.47	21.43	–

*n* = number of cases. If n is not indicated, all parents of the group replied to the question. Numbers in the table represent percentages.

a Refugee = both parents refugee parents. Recently = recently immigrated parents. Immig. = Immigrant-background (either one of the parents born abroad or at least one of the parents having parents which were born abroad). Non-immig. = Non-immigrant parents.

b Syria, Iraq, Lebanon, Morocco.

c Bulgaria, France, Kosovo, Macedonia, Netherlands, Poland, Russia, Serbia, Turkey, Ukraine.

d Ghana, India, Israel, Kazakhstan, Kyrgyztan, Nigeria, Pakistan, Sudan.

**Table 3:** Descriptives on children.

Descriptives		Refugee^a^	Recently^a^	Immig.^a^	Non-imm.^a^
Gender	Female	47.83	45.45	42.86	40.91
Current grade visited	Three	26.09	13.63	17.14	-
	Four	73.91	86.36	80.00	100.00
Country of birth	Germany	13.04	68.18	100.00	100.00
	Arab countries^b^	86.96	4.54	-	-
	Other	-	27.28	-	-
Early childcare attendance	Yes, Germany	39.13	72.72	75.00	72.73
	Yes, abroad	30.43	27.27	17.85	27.27
School enrollment in Germany	Yes	91.30	95.45	100.00	100.00
Childcare attendance after school	Never	-	-	-	-
	1–2 days	-	15.38	25.00	14.29
	3–5 days	100.00	84.62	75.00	85.71

All parents answered to the question. Numbers in the table represent percentages.

a Refugee = both parents refugee parents. Recently = recently immigrated parents. Immig. = Immigrant-background (either one of the parents born abroad or at least one of the parents having parents which were born abroad). Non-immig. = Non-immigrant parents.

b Syria, Iraq, Lebanon, Morocco.

### 2.2 Measurement

#### 2.2.1 Parents’ academic engagement and resources

We based the home and school engagement scales on an extensive literature search (e.g., Bakker et al., 2007; Khawaja et al., 2017; Kohl et al., 2000; Ravens-Sieberer and Bullinger, 1998; Turney and Kao, 2009). However, a widely accepted measure for parents’ academic engagement did not exist (Turney and Kao, 2009). When looking at much-used scales (e.g., Bakker et al., 2007; Khawaja et al., 2017; Kohl et al., 2000; Ravens-Sieberer and Bullinger, 1998), these items and their wording did not fit well to RRI parents. Existing scales (a) did not cover the aspects relevant to RRI and immigrant-background parents’ academic engagement in Germany; (b) included some culturally non-applicable or, for our purposes, outdated items (e.g., referring to old technology, not taking COVID-related school closures into account); or (c) used too complicated wording. Therefore, based on our literature research and consultations with practitioners, we developed own items which incorporated concepts of previous studies, yet were most suitable for (recently arrived) refugee and immigrant-background parents living within contexts in lower-income neighborhoods in Germany. We conducted a pilot study with eight Turkish immigrant-background and Arabic-speaking refugee parents in the respective languages. Subsequently, we consulted these parents about the items and open-format questions to increase the validity of our interview. As all items seemed understandable to the parents and appeared to measure those behaviors and resources that we intended to measure, we did not need to delete or adjust items. The developed items appeared to yield better applicability and relevance for our study purpose (i.e., participants understood the meaning of the questions and items related to their specific living circumstances). All *resources* and *engagement* items can be found in Table 4. For measuring parents’ *formal education*, we created a 5-point scale indicating whether the parents received only primary education (up to six years); (1), partly lower secondary education (2), lower secondary education (at least 10 years of school); (3), upper secondary education (4) or tertiary education (5). For *language skills* and *education,* we used mean scores of both partners (or the single parent), as all engagement questions asked for either the engagement of the interviewee or the partner. Thus, the measure of language skills indicated the interviewees’ and their partners’ abilities to understand written and oral information in the school context. The *home engagement*, *aspirations*, and *health* scales yielded too low McDonald’s *ω*s, implying that the respective items did not measure homogenous constructs. Thus, we used two single indicator items for reading with the child/child reads by him−/herself and going on outings (e.g., park, playground, library, zoo) with the child, as well as a four-item homework-engagement scale (for detail, see Table 4). We examined the use of single items for the different aspects of homework engagement (e.g., supporting and controlling homework) based on our data. Nonetheless, McDonald’s *ω*s did not indicate an improvement if any item was to be excluded. We also correlated the single homework items with predictors of RQ1 and outcomes of RQ2. We found no indicators for different influences of the single items and thus used all homework items as a scale. We also display correlations of the single homework items in Table 5. Thus, we opted for the whole scale as a more reliable approach. The *structural learning environment* score was built with the sum score of four dichotomous items (availability of a quiet room in the apartment, availability of own desk, availability of WiFi or cable-bound internet in the apartment, the possibility of using a tablet or computer for learning).

**Table 4:** Information on scales, constructs, and items.

Item	*ω*	Study^a^
Home engagement—educational activities at home (never or seldom—daily)		
1. Do you ask your child about her/his day in school when she/he comes home?	0.19	dropped, lv
2. Do you have joined meals with your child?		dropped, lv
3. Do you read with your child or does she/he read alone?		single
4. Do you go on outings with your child, e.g., to the zoo, park, museum, or library?		single
Home engagement—homework engagement (never or seldom—daily)	0.67	used as scale
1. How often is a family member aware of what the child learns in school?		
2. How often does a family member check that the child has done her/his homework?		
3. How often is a family member available to help the child with homework?		
4. How often does a family member do homework together with the child?		
School engagement (not yet—every time) * (not true—true)		used as scale
1. How often do you know about new information that is passed in school, e.g., that there will be a parent meeting or school event?	0.75	
2. How often do you or your partner attend teacher-parent meetings?		
3. How often do you or your partner attend parent council meetings?		
4. How often do you or your partner help out at school activities (e.g., an event, outing or working group)?		
5. How often do you have contact with the class teacher?		
6. How often do you have contact with other parents in the school?		
7. It is easy for me to get in contact with other parents at the school.*		
8. If COVID-19 was over, would you or your partner like to be engaged in school?*		
Parents’ aspirations (not true—true)		
1. It is important for me that my child has good grades.	0.25	single
2. It is important for me that my child will have a high income.		single
3. It is important for me that my child learns a lot in school.		dropped, lv
Resources—parents’ mental health (not true—true; original scale: The WHOQOL Group, 1998)		used as scale
1. I am satisfied with my health.	0.57	orig, dropped
2. I am often stressed in daily life.		
3. I often have negative feelings such as sadness, despair, anxiety, or depressiveness.	*r* = 0.45	orig
Resources—parents’ language skills (asked for interviewee/partner; not true—true)		used as scale
1. I can effortlessly communicate with the class teacher in German.	*r* = 0.73, 0.66^b^	
2. I understand German e-mails and flyers from the school.		
Resources—parents’ social support (not true—true)		
1. I regularly have contact with family or friends that live close by.	0.55	dropped, lv
2. I have someone that can take care of my child if I have, e.g., a spontaneous appointment.		single
3. I have a person I can share my joy and worries with.		dropped, lv
4. I regularly attend a local social support group, e.g., a parent or religious group.		single
Resources—parents’ knowledge of the education system (not true—true)		
1. I know the differences between the different school types in Germany and I know which school leaving qualifications are required for different professions.		single
Resources—parent-teacher relationship (not true—true)		used as scale
1. I enjoy talking to the class teacher.	0.69	
2. The class teacher pays attention to my suggestions and concerns.		
3. It is easy for me to get in contact with the class teacher.		
Resources—parents’ sense of wellbeing at school (not true—true)		
1. I feel comfortable at this school.		single
Child well-being at school (not true—true; original scale: Ravens-Sieberer and Bullinger, 1998)		used as scale
1. My child feels comfortable at school.	0.70	
2. My child enjoys lessons.		orig
3. My child is afraid of bad grades.		orig
4. School is exhausting for my child.		
5. My child’s behavior or emotions in school attract negative attention.		
6. My child manages tasks in school well.		orig

ω = McDonald’s Omega (used as reliability measure).

a Indicates how the item was used in this study. Dropped = not used in this study, single = used as a single scale item (i.e., because *ω* of the entire scale was too low), lv = there was only little variance in parents’ replies to the item, orig = We used the exact item of the original scale.

b Partner. * (not true—true).

**Table 5:** Correlations of the outcome variables and all predictors (RQ2).

Predictors	Hyperactivity/inattention	Emotional symptoms	Prosocial behavior	Well-being at school	Grades
Homework engagement	0.06	−0.18	0.19	0.06	0.07
a. Knowing content	−0.05	−0.26	0.06	0.28	0.23
b. Control homework	0.06	−0.09	0.05	0.00	0.01
c. Be available to help with	0.10	−0.16	0.24	0.13	0.01
d. Do homework together	0.04	−0.04	0.17	−0.11	0.02
School engagement	−0.15	−0.05	−0.06	0.04	0.09
Reading with the child	−0.17	−0.12	0.05	0.08	0.23
Going on outings with the child	−0.27	0.02	−0.17	−0.12	0.21

We used Pearson’s correlation coefficient *r*.

All scale items were measured on 5-point Likert scales reaching from 0 to 4. For frequencies, the scale reached from “0 = never or seldom,” “1 = several times during the month (2-3x per month),” “2 = once a week,” “3 = 2–4 times per week,” to 4 = “every day.” Our goal was to design the scales as simple as possible, due to infrequent events in schools, however, we adjusted the scale for school engagement questions to “0 = not yet,” “1 = one time,” “2 = more than one time,” “3 = almost every time,” and “4 = every time.” For sentiments and opinions, we used a scale reaching from “0 = not true (no),” “1 = often not true (rather no),” “2 = sometimes yes, sometimes no,” “3 = often true (rather yes),” = to “4 = true (yes).” We facilitated parents’ responses by using simple wording and providing visualizations of the scales before the interview (sent to the parents by letter mail). Table 4 shows all constructs, scales, items, McDonald’s *ω*s (internal consistency), and the process of item selection. Means and standard deviations of parent academic engagement and resource variables can be found in Table 6.

**Table 6:** Means and standard deviations of parent constructs and items.

Constructs and items	Refugee^a^		Recentlys^a^		Immig.^a^		Non-imm.^a^		
	*M*	*SD*	*M*	*SD*	*M*	*SD*	*M*	*SD*	*Range*
Homework engagement	12.61	3.35	12.32	2.93	12.00	3.30	12.64	3.21	0–16
School engagement	**21.17**	**4.49**	23.36	3.29	24.50	3.42	24.55	3.36	0–32
Reading with the child	2.17	1.37	2.59	1.40	2.71	1.21	2.73	0.93	0–4
Going on outings w/ the child	1.61	0.84	1.68	1.04	1.46	0.88	1.45	1.06	0–36
Aspirations: good grades	3.48	1.20	3.27	1.28	3.29	0.94	3.55	0.67	0–4
Aspirations: high income	2.87	1.42	2.91	1.63	2.43	1.45	2.36	1.43	0–4
Formal education^b^	3.11	1.32	3.64	1.01	3.41	0.62	3.43	0.79	1–5
Mental health	**4.00**	**2.32**	4.45	2.86	**4.14**	**2.01**	5.86	1.13	0–8
Language skills^b^	**3.07**	**0.86**	**2.83**	**1.35**	**3.87**	**0.31**	4.00	0.00	0–4
Support: person	**2.61**	**1.70**	**2.91**	**1.60**	3.68	0.55	3.55	0.96	0–4
Support: social group	1.61	1.70	**2.23**	**1.82**	**2.21**	**1.69**	0.73	1.24	0–4
Knowledge of edu system	**3.00**	**1.28**	3.50	1.06	3.93	0.38	3.77	0.61	0–4
Parent-teacher relationship	10.35	1.94	10.95	1.43	11.25	0.89	10.18	2.50	0–12
Sense of well-being at school	3.09	1.20	3.32	0.89	3.39	1.10	3.09	1.44	0–4
Structural learning environm.	3.57	0.66	3.59	0.73	3.59	0.66	3.91	0.29	0–4

*M* = mean. *SD* = standard deviation. *Range* = possible range. Values in bold significantly differed between groups.

a Refugee = both parents refugee parents. First-gen. = recently immigrated (both parents born abroad, but not refugee). Immig. = Immigrant-background (either one of the parents born abroad or at least one of the parents having parents which were born abroad). Non-imm. = Non-immigrant parents.

b Mean of interviewee’s and partner’s values.

#### 2.2.2 Children’s school adjustment

Children’s socioemotional adjustment was measured with the German adaptation of the parent version of the Strengths and Difficulties Questionnaire (SDQ; Goodman, 1997). McDonald’s *ωs* were unacceptable for conduct problems (*ω* = 0.53) and peer problems (*ω* = 0.57). Thus, we only analyzed the subscales hyperactivity/inattention (*ω* = 0.78), emotional symptoms (*ω* = 0.71), and prosocial behavior (*ω* = 0.70). For measuring *children’s grades*, we created a sum score consisting of children’s German and Math grades of the past term. The German grade system ranges from 1 to 6, with 1 being the best grade. For better interpretability, we reverse-coded the sum score. Means and standard deviations of children’s school adjustment variables can be found in Table 7.

**Table 7:** Means and standard deviations of child constructs and items.

Constructs and items	Refugee^a^		Recently		Immig.		Non-imm.		
	*M*	*SD*	*M*	*SD*	*M*	*SD*	*M*	*SD*	*Range*
Hyperactivity/inattention	3.86	2.25	3.70	2.05	3.52	2.86	4.50	3.19	0–10
Emotional symptoms	**3.05**	**2.13**	2.75	2.65	2.17	2.35	1.27	1.55	0–10
Prosocial behavior	9.09	1.41	9.30	1.17	9.00	1.31	9.05	1.70	0–10
Well-being at school	17.17	3.74	18.23	4.16	18.64	4.21	17.23	4.06	0–24
Grades^b^	9.52	1.53	9.00	2.05	9.00	1.27	8.67	1.49	2–12

*M* = mean. SD = standard deviation. Range = possible range. Values in bold significantly differed between groups.

a Refugee = both parents refugee parents. First-gen. = recently immigrated (both parents born abroad, but not refugee). Immig. = Immigrant-background (either one of the parents born abroad or at least one of the parents having parents which were born abroad). Non-imm. = Non-immigrant parents.

b Comprised of German and Math grades. In the German education system, grades reach from 1 (best) to 6 (worst). We inverse-coded grades, thus, 12 is the best possible grade score.

#### 2.2.3 Open-ended questions

In open-ended questions (see Table 8), we asked for details about the examined constructs. The topics included: (a) homework engagement, (b) perceived responsibilities in their children’s education, (c) extent and manner of contact with the class teacher and other parents at school, (d) reasons for parents’ academic engagement, (e) parents’ educational aspirations for their children, and (f) parents’ stressors in daily life. Parents’ replies were later summarized into categories, a common procedure for research with refugees (e.g., Busch et al., 2018; Rah et al., 2009). Therefore, we summed the number of parents who gave the specific reply.

**Table 8:** Parents’ replies to open ended question (categorized).

Parents’ replies	Refugee	Recently	Immigrant	Non-imm.
Home engagement: Which places did you or your partner already visit with your child? (*n* = 22, 22, 27, 22)				
Listed 4 or more places	18.18	54.55	40.74	72.73
Listed 2–3 places	54.54	40.90	55.56	27.27
Listed 0–1 places	31.82	4.55	3.70	–
Home engagement: Where does your child do homework? (*n* = 23, 22, 27, 22)				
At home	65.22	72.73	76.92	77.27
At school/childcare facility/tutoring/other	69.57	59.09	30.77	45.45
Home engagement: Who checks the child’s homework? (*n* = 23, 22, 28, 22)				
Interviewee/partner	91.30	95.45	82.14	90.91
Siblings/relatives/teacher/other	08.70	31.82	28.57	9.09
No one	–	–	7.14	4.55
Home engagement: Who does the homework together with the child? (*n* = 23, 22, 28, 22)				
Interviewee/partner	69.57	86.36	89.29	81.82
Siblings/relatives/teacher/other	21.74	40.91	35.71	36.36
No one	13.04	–	7.14	13.64
Home/school engagement: Who is responsible for the child’s learning? The school or the parents? (*n* = 23, 22, 28, 22)				
Parents	8.70	13.63	25.00	9.09
School	47.83	18.18	21.43	13.64
Both	43.48	68.18	53.57	77.27
Home/school engagement: What are the parent’s tasks? (*n* = 23, 21, 28, 22)				
helping with homework/learning	21.74	42.86	28.57	63.64
Checking homework/acquired knowledge	26.09	57.14	35.71	36.36
Providing material/environment	4.35	14.29	3.57	18.18
Keep contact with the teacher	8.70	14.29	7.14	-
Make sure the child behaves in school	-	4.76	7.14	9.09
Motivate child, emotional support	8.70	38.10	22.86	25.00
School is responsible (e.g., language issues)	26.09	-	5.71	-
School engagement: How do you receive information from the school? (*n* = 23, 22, 28, 22)				
Other parents/other person/WhatsApp	17.39	27.27	28.57	40.91
Written communication (flyer/e-mails/app)	60.87	77.27	78.57	81.82
Direct contact with teacher	39.13	27.27	14.29	9.09
School engagement: Why is it (not) easy for you to get in contact with other parents at this school? (*n* = 20, 20, 23, 21)				
Other parents are friendly/already knowing parents	5.00	25.00	34.78	9.52
Parent enjoys socializing	–	5.00	17.39	47.62
Sharing same cultural background or language	10.00	10.00	4.35	–
Contact through WhatsApp group, telephone	15.00	20.00	34.78	9.52
Parent does not wish to socialize	15.00	5.00	–	4.76
Difficult—language barrier	40.00	20.00	–	9.52
Difficult—others do not want to talk, discrimination, lack of socializing possibilities (Covid-19)	25.00	15.00	4.35	9.52
Who initiates the contact with the class teacher? (*n* = 15, 21, 28, 22)				
Parents	53.33	23.81	39.29	09.09
Class teacher	33.33	9.52	19.05	13.64
Both	13.33	66.66	46.43	77.27
School engagement: If Covid-19 was over, why would you (not) like to be engaged in school? (*n* = 17, 17, 23, 22)				
Supporting the child	88.24	58.82	78.26	40.91
Staying informed, it is important	17.65	11.76	17.39	27.27
It is enjoyable	–	11.76	–	9.09
Contact with teacher/other parents	11.76	11.76	17.39	4.44
Difficult—time constraints	17.65	–	4.35	13.64
Aspirations: Do you wish for your child to find a job right after school, do an apprenticeship, study for a degree, or something else? (*n* = 23, 22, 28, 22)				
Apprenticeship	–	18.18	25.00	31.82
Studies/degree	95.65	40.91	32.14	18.18
unimportant	4.35	36.36	39.29	50.00
Mental health: In your daily life, are there stressful situations? Can you name an example? (*n* = 20, 20, 26, 22)				
Caring for children/responsibility	45.00	20.00	26.92	18.18
Own or family member illness	–	15.00	11.54	4.55
Time constraints/work/too many appointments	35.00	35.00	34.62	36.36
Language barriers	20.00	10.00	–	–
Discrimination/acculturation difficulties	15.00	15.00	–	–
Not many problems	10.00	25.00	19.24	31.32

Numbers in the table represent percentages. We only listed topics that were mentioned by at least five parents across groups. Refugee = both parents refugee parents. Recently = recently immigrated (both parents born abroad, but not refugee). Immigrant = Immigrant-background (either one of the parents born abroad or at least one of the parents having parents which were born abroad). Non-imm. = Non immigrant parents.

### 2.3 Statistical analysis

We conducted all analyses in *RStudio* based on *R 4.1.2* using packages *car, FSA, GPArotation, Hmisc,* and *psych.* For RQ1 and RQ2, we used multiple linear regression modeling. We used the functions *par, plot,* and *vif* to examine assumptions for linear modeling. We used the package *psych* for correlation analyses. We conducted one-way *ANOVA*s and *t-*tests to detect group differences in RQ3. If assumptions were violated, we used *Kruskal-Wallis H tests* and *Dunn tests*. For RQ4, we screened the parents’ replies to open-ended questions and determined the percentages of categories for each question mentioned by at least five parents across groups. We additionally analyzed some single items from the scales (see Table 4) with either one-way *ANOVAs* and pairwise *t*-tests or *Kruskal-Wallis H tests* and *Dunn tests*.

## 3 Results

### 3.1 Preliminary analysis

Due to the explorative scope of our study and little previous work available involving the specific sample of our study, we opted to include all resource predictors. Thus, we reduced the possibility that some effects might be masked by other variables. In *a priori t*-tests, non-immigrant parents differed from RRI and non-immigrant parents regarding their health, German language skills, attendance of local social support groups, and their children’s emotional symptoms. Thus, we excluded the *n* = 22 non-immigrant parents from the analyses of RQ1 and RQ2.

### 3.2 Determinants of parents’ academic engagement (RQ1)

Results of the multiple linear regression analyses predicting parents’ homework engagement, school engagement, reading with the child, and going on outings with the child can be found in Table 9. The regression on reading with the child just reached non-significance (*p* = 0.051), but the predictors knowledge of the education system (*p* = 0.041) and the value parents placed on their children’s grades (*p* = 0.044) reached significance. Language skills (*p* = 0.074) did not become significant yet reached small *p*-value. The regression on going on outings with the child was significant (*p* = 0.032), with parents’ German language skills negatively predicting the extent to which they went on outings with the child (*p* = 0.007) and the extent of attending local social support groups positively predicting it (*p* = 0.001). All other predictors were non-significant. The regression on homework engagement was non-significant (*p* = 0.846). The regression on parents’ school engagement was significant (*p* = 0.037), with parents’ German language skills (*p* = 0.045) and the extent of attending local social support groups (*p* = 0.041) being positive predictors. The parent-teacher relationship (*p* = 0.071) did not become significant but reached small *p*-values. Additionally, we provide correlations of all predictors, including single homework items, with the outcome variables in Table 10.

**Table 9:** Multiple regression analysis predicting parents’ homework and school engagement.

Regression Estimates							
	*b*	*SE*	*ß*	*T*	*p*	*KI(l)*	*KI(u)*
~ Reading with the child							
Aspirations: good grades	0.261	0.128	0.291	2.044	0.044*	0.007	0.516
Aspirations: high income	−0.486	0.090	−0.072	−0.541	0.590	−0.228	0.130
Health	0.046	0.058	0.113	0.792	0.431	−0.070	0.163
Formal education	0.178	0.142	0.176	1.248	0.216	−0.106	0.462
Language skills	0.258	0.142	0.245	1.812	0.074	−0.026	0.541
Support: having someone near	−0.092	0.108	−0.123	−0.856	0.395	−0.307	0.123
Support: local social support group	−0.064	0.078	−0.111	−0.817	0.416	−0.218	0.091
Knowledge of the education system	0.310	0.149	0.300	2.084	0.041*	0.014	0.607
Parent-teacher relationship	0.090	0.096	0.131	0.947	0.347	−0.100	0.281
Sense of well-being at school	−0.096	0.137	−0.098	−0.699	0.487	−0.369	0.177
*R^2^* = 0.207, R*^2^_adj_* = 0.100, *F*(10,75) = 1.954*, p* = 0.051							
~ Going on outings with the child							
Aspirations: good grades	0.006	0.090	0.006	0.062	0.951	−0.174	0.185
Aspirations: high income	0.005	0.063	0.007	0.077	0.938	−0.122	0.131
Health	−0.032	0.041	−0.078	−0.777	0.440	−0.114	0.050
Formal education	−0.048	0.101	0.047	0.474	0.637	−0.153	0.248
Language skills	**−0.279**	**0.100**	**−0.265**	**−2.777**	0.007**	**−0.479**	**−0.079**
Support: having someone near	−0.023	0.076	−0.031	−0.302	0.763	−0.175	0.129
Support: local social support group	**0.187**	**0.055**	**0.325**	**3.399**	0.001**	**0.077**	**0.296**
Knowledge of the education system	0.072	0.105	0.070	0.688	0.493	−0.137	0.282
Parent-teacher relationship	−0.004	0.067	−0.006	−0.061	0.951	−0.139	0.130
Sense of well-being at school	0.024	0.097	0.247	0.249	0.804	−0.169	0.217
*R^2^* = 0.222*, R^2^_adj_* = 0.118, *F*(10,75) = 2.136*, p* = 0.032							
~ Parents’ homework engagement							
Aspirations: good grades	−0.014	0.347	−0.016	−0.040	0.968	−0.706	0.676
Aspirations: high income	0.121	0.244	0.179	0.496	0.621	−0.365	0.607
Health	0.137	0.159	0.335	0.863	0.391	−0.179	0.453
Formal education	−0.408	0.387	0.403	−1.054	0.295	−1.179	0.363
Language skills	0.276	0.386	0.259	0.706	0.482	0.496	1.041
Support: having someone near	−0.109	0.293	−0.145	−0.371	0.712	−0.693	0.475
Support: local social support group	0.304	0.211	0.530	1.440	0.154	−0.116	0.725
Knowledge of the education system	−0.258	0.404	−0.250	−0.639	0.525	−1.064	0.547
Parent-teacher relationship	0.139	0.259	0.203	0.537	0.593	−0.377	0.656
Sense of well-being at school	−0.145	0.372	−0.149	−0.391	0.697	−0.886	0.596
*R^2^* = 0.069, *R^2^_adj_* = −0.055, *F*(10,75) = 0.554*, p* = 0.846							
~ Parents’ school engagement							
Aspirations: good grades	−0.420	0.425	−0.469	−0.988	0.326	−1.266	0.426
Aspirations: high income	−0.108	0.299	−0.160	−0.361	0.719	−0.703	0.487
Health	0.201	0.194	0.491	1.034	0.304	−0.186	0.588
Formal education	0.056	0.473	0.057	0.122	0.904	−0.886	1.001
Language skills	**0.963**	**0.472**	**0.915**	**2.039**	0.045*	**0.022**	**1.903**
Support: having someone near	−0.013	0.359	−0.017	−0.036	0.971	−0.728	0.702
Support: local social support group	**0.538**	**0.258**	**0.938**	**2.084**	0.041*	**0.024**	**1.053**
Knowledge of the education system	0.843	0.495	0.815	1.705	0.092	−0.142	1.829
Parent-teacher relationship	0.581	0.317	0.844	1.830	0.071	−0.051	1.213
Sense of well-being at school	0.018	0.455	0.018	0.039	0.970	−0.889	0.924
*R^2^ = 0.217, R^2^_adj_* = 0.112, *F*(10,75) = 2.076*, p* = 0.037							

*b* = unstandardized regression coefficient. *SE* = standard error of *b*. *ß* = standardized regression coefficient. *T* = *t*-value. *p* = two-tailed *p*-values. *KI(l)* = 2.5%, *KI(u)* = 97.5%. *R*^2^ = Multiple R-squared. *R^2^_adj_* = adjusted *R*^2^.

**Table 10:** Correlations of the outcome variables and all predictors as well as structural learning environment (RQ1).

Predictors	Homework engagement	School engagement	Reading with the child	Going on outings w/ the child
Aspirations—good grades	0.04	−0.04	0.24	0.08
Aspirations—high income	0.05	−0.08	−0.04	−0.04
Formal education	−0.07	0.05	0.12	0.08
Mental health	−0.09	−0.18	−0.14	0.07
Language skills	0.10	0.29	0.19	−0.29
Support—person	−0.02	0.09	−0.01	−0.07
Support—social group	0.17	0.20	−0.04	0.34
Knowledge of education system^a^	−0.07	0.21	0.25	0.00
Parent-teacher relationship	0.02	0.21	0.21	−0.06
Sense of well-being at school	−0.04	0.08	0.02	−0.05
Structural learning environment	0.04	0.03	0.19	0.08

a Knowledge of the German education system. We used Pearson’s correlation coefficient *r*.

### 3.3 Linking parents’ academic engagement to children’s school adjustment (RQ2)

Results of the multiple linear regression analyses predicting the children’s school adjustment with the academic engagement variables can be found in Table 11. Albeit the regression on hyperactivity/inattention was non-significant (*p* = 0.072), the predictor going on outings reached significance (*p* = 0.029). The regressions on emotional symptoms (*p* = 0.512), on prosocial behavior (*p* = 0.246), on well-being at school (*p* = 0.673), and on grades (*p* = 0.128) were non-significant. Additionally, we provide correlations of all predictors, including single homework items, with the outcomes in Table 5.

**Table 11:** Multiple regression analysis predicting child outcomes.

Regression Estimates							
	*b*	*SE*	*ß*	*T*	*p*	*CI(l)*	*CI(u)*
~ Hyperactivity/inattention							
Reading with the child	−0.242	1.882	−0.305	−1.109	0.271	−0.676	0.193
Going on outings with the child	−0.643	0.218	−0.578	−2.233	0.029*	−1.216	−0.069
Homework engagement	0.051	0.086	0.159	0.585	0.561	−0.121	0.222
School engagement	−0.062	0.064	−0.260	−0.968	0.336	−0.189	0.065
*R^2^* = 0.111*, R^2^_adj_* = 0.062, *F*(4,72) = 2.249*, p* = 0.072							
~ Emotional symptoms							
Reading with the child	−0.177	0.216	−0.224	−0.823	0.413	−0.608	0.253
Going on outings with the child	0.084	0.285	0.075	0.294	0.770	−0.484	0.652
Homework engagement	−0.123	0.085	−0.390	−1.447	0.152	−0.293	0.047
School engagement	−0.019	0.063	−0.080	−0.299	0.766	−0.145	0.107
*R^2^* = 0.044*, R^2^_adj_* = −0.009, *F*(4,72) = 0.827*, p* = 0.512							
~ Prosocial behavior							
Reading with the child	0.067	0.119	0.084	0.562	0.576	−0.170	0.304
Going on outings with the child	−0.246	0.157	−0.221	−1.564	0.122	−0.559	0.067
Homework engagement	0.073	0.047	0.232	1.562	0.123	−0.020	0.167
School engagement	−0.017	0.035	−0.069	−0.472	0.638	−0.086	0.053
*R^2^* = 0.072*, R^2^_adj_* = −0.020, *F*(4,72) = 1.390*, p* = 0.246							
~ Well-being at school							
Reading with the child	0.281	0.353	*0.281*	0.795	0.429	−0.422	0.983
Going on outings with the child	−0.589	0.484	*−0.589*	−1.218	0.227	−1.552	0.373
Homework engagement	0.061	0.139	*0.061*	0.437	0.663	−0.215	0.336
School engagement	−0.029	0.104	*0.029*	0.275	0.784	−0.178	0.236
*R^2^ = 0.028, R^2^_adj_* = −0.020, *F*(4,81) = 0.586*, p* = 0.673							
~ Grades							
Reading with the child	0.243	0.150	0.306	1.614	0.111	−0.057	0.543
Going on outings with the child	0.325	0.213	0.292	1.522	0.132	−0.100	0.750
Homework engagement	0.030	0.058	0.094	0.508	0.613	−0.087	0.146
School engagement	0.022	0.045	0.095	0.504	0.616	−0.066	0.107
*R^2^* = 0.089*, R^2^_adj_* = 0.041, *F*(3,77) = 1.852*, p* = 0.128							

*b* = unstandardized regression coefficient. *SE* = standard error of *b*. *ß* = standardized regression coefficient. *T* = *t*-value. *p* = two-tailed *p*-values. *KI(l)* = 2.5%, *KI(u)* = 97.5%. *R*^2^ = Multiple R-squared. *R^2^_adj_* = adjusted *R*^2^. Values printed in bold indicate significant differences between groups.

### 3.4 Group differences in parent and child variables (RQ3)

Tables 6, 7 display descriptive statistics of the variables and group differences. Refugee parents reported lower school engagement than non-immigrant parents (*t* = 2.841, *p* = 0.007) and immigrant-background parents (*t* = 3.005, *p* = 0.004), *F*(3, 90) = [4.301], *p* = 0.007. The difference between refugee and recently immigrated parents was close to being significant (*t* = 1.850, *p* = 0.070). Non-immigrant parents reported better mental health than refugee parents (*z* = 2.844, *p* = 0.004) and immigrant-background parents (*z* = 3.045, *p* = 0.002), *χ*^2^(3) = 11.396, *p* = 0.009. Refugee and recently immigrated parents both reported lower German language skills than non-immigrant parents (*refugee: z* = 4.485, *p* < 0.001; *recently immigrated*: *z* = 4.459, *p* < 0.001) and immigrant-background parents (*refugee: z* = 3.828, *p* < 0.001; *recently immigrated: z* = 3.695, *p* < 0.001), *χ*^2^(3) = 34.770, *p* < 0.001. Fewer refugee parents had local social support (*person able to support*) than non-immigrant parents (*t* = 2.262, *p* = 0.029) and parents with an immigrant-background (*t* = 3.145, *p* = 0.002), *F*(3, 90) = [4.269], *p* = 0.007. Recently immigrated parents also reported less local social support (*person able to support*) than immigrant-background parents (*t* = 2.378, *p* = 0.021). However, non-immigrant parents attended fewer local social support groups than immigrant-background parents, (*t* = −3.462, *p* = 0.001), and recently immigrated parents, (*t* = −3.188, *p* = 0.003), *F*(3, 90) = [4.349], *p* = 0.007. Refugee parents reported a significantly lower knowledge of the education system than non-immigrant parents, (*z* = 3.107, *p* = 0.002), parents with an immigrant-background, (*z* = 4.063, *p* < 0.001), and recently immigrated parents, (*z* = 2.150, *p* = 0.032), *χ*^2^(3) = 17.909, *p* < 0.001. Refugee (*z* = −3.026, *p* = 0.002) and recently immigrated parents (*z* = −2.028, *p* = 0.043) also reported their children to have significantly more emotional symptoms than non-immigrant children, *χ*^2^(3) = 9.835, *p* = 0.020.

We found no differences between groups regarding homework engagement, *χ*^2^(3) = 1.357, *p* = 0.716, reading with the child, *F*(3, 90) = [0.676], *p* = 0.569, going on outings with the child, *F*(3, 90) = [0.298], *p* = 0.827, value placed on the children’s grades, *χ*^2^(3) = 1.805, *p* = 0.614, value placed on the child’s future income, *χ*^2^(3) = 4.088, *p* = 0.252, parents’ well-being at school, *χ*^2^(3) = 1.233, *p* = 0.745, parent-teacher relationship, *χ*^2^(3) = 2.206, *p* = 0.531, parents’ formal education, *χ*^2^(3) = 2.747, *p* = 0.432, children’s hyperactivity/inattention, *χ*^2^(3) = 1.243, *p* = 0.743, prosocial behavior, *χ*^2^(3) = 0.925, *p* = 0.819, children’s well-being at school, *F*(3, 90) = [0.812], *p* = 0.491 or children’s grades, *χ*^2^(3) = 3.849, *p* = 0.278.

Across all groups, we also found several indicators for a progressed integration process as well as that parents were highly willing to support their children’s education. We found high rates for early childcare attendance (91.67% of all children) and school enrollment in Germany since first grade (97.22% of all children). Additionally, almost all parents fully agreed to the statement that they daily asked their children about their school day (*M* = 3.83, *Mdn* = 4.00, *SD* = 0.46) and that they had daily meals with their children (*M* = 3.75, *Mdn* = 4.00, *SD* = 0.60).

### 3.5 Exploring barriers and facilitators to parents’ academic engagement (RQ4)

Table 8 shows an overview of the percentages of replies to the open-ended questions. Here, we will outline the most important responses to open-ended questions on a descriptive level. We also report some descriptive statistics of single-scale items as well as group comparisons of those.

#### 3.5.1 Parents’ academic engagement and resources

When refugee parents went on outings with their children, they had yet taken them to fewer different places than parents of the other groups (18.18% of *refugee* parents named four or more places, whereas 54.55% of *recently immigrated*, 40.74% of *immigrant-background*, and 72.73% of *non-immigrant* did; 31.82% of *refugee* parents also listed only 0–1 places, whereas less than 5% of all other groups did). Noticeably, *recently immigrated* parents named fewer places than *non-immigrant parents*, but more *than immigrant-background* parents.

65.22% of *refugee*, 72.73% of *recently immigrated,* 76.92% of *immigrant-background*, and 77.27% of *non-immigrant* children did their homework, at least partly, at home. On a descriptive level, refugee children did their homework less often together with their parents, relatives, or teachers than children of the other groups. Almost all parents fully agreed to the statement that they would like to be engaged in school, if possible (*M* = 3.64–3.86, *Mdn* = 4, *SD* = 0.35–0.95). Main reasons were the desire to support their children, to a lesser extent also to have contact with the teacher or other parents, and to stay informed. Only a few parents perceived time constraints as a barrier to engagement, e.g., due to work or other colliding responsibilities such as childcare. However, more than one-third of all groups considered time constraints frequent stressors in daily life. Other frequent stressors for some RRI parents were responsibility for their children and household chores (45.00% *refugee,* 20.00% *recently immigrated*), language barriers (20.00% *refugee*, 10.00% *recently immigrated*), and discrimination or acculturation difficulties (15.00% *refugee*, 15.00% *recently immigrated*). On a descriptive level, refugee parents more often wanted their children to obtain a university or college degree after school (95.65% vs. 40.91% *recently immigrated*, 32.14% *immigrant-background*, and 18.18% *non-immigrant*). Of all parents, they also more often saw the sole responsibility for their children’s learning with the school (47.83% *refugee* vs. 18.18% *recently immigrated*, 21.43% *immigrant-background*, 13.64% *non-immigrant*).

Most of the parents considered helping with homework or learning, checking the homework, supporting their child emotionally, and providing an adequate environment and material their most important tasks in their children’s education. When descriptively comparing refugee parents to other parents, they less often saw their responsibilities in motivating their children, checking homework, or helping with homework. Instead, about one-fourth of them saw the responsibility with the school, mostly caused by language barriers. Recently immigrated parents more often saw their responsibilities in motivating their children and checking their homework than the other groups.

#### 3.5.2 Contact with the school

Most parents received information from the school via written information such as leaflets, e-mails, or a school app (60.87% *refugee,* 77.27% *recently immigrated*, 78.57% *immigrant-background*, 81.82% *non-immigrant*). Additionally, especially refugee (39.13%) and to a lesser extent recently immigrated (27.27%) parents received information directly from the teacher (vs. 14.29% *immigrant-background*, 9.09% *non-immigrant*).

Surprisingly, only a few parents (8.70% *refugee*, 14.29% *recently immigrated*, 7.14% *immigrant-background*) considered it their task to stay in contact with the class teacher. Almost all parents fully agreed to the statement that it was easy for them to get in contact with the class teacher (*M* = 3.64–3.86, *Mdn* = 4, *SD* = 0.45–0.73). There were no group differences in the frequency of having contact with the class teacher, *χ*^2^(3) = 3.6292, *p* = 0.304. On a descriptive level, *refugee* parents (53.33%) more often initiated contact with the class teacher than *recently immigrated* (23.81%), *immigrant* (39.29%), and *non-immigrant* parents (9.09%). Thus, *immigrant-background* parents also more often initiated contact with the class teacher than *non-immigrant* parents on a descriptive level.

#### 3.5.3 Contact with other parents

Additionally, it was more difficult for *refugee* parents to get in contact with other parents (*M* = 2.00, *SD* = 1.38) than it was for *non-immigrant* parents (*M* = 3.41, *SD* = 1.10, *z* = 3.787, *p* < 0.001), parents with an *immigrant-background* (*M* = 3.71, SD = 0.53, *z* = 4.687, *p* < 0.001) and *recently immigrated* parents (*M* = 2.77, *SD* = 1.60, *z* = 2.965, *p* = 0.014), *χ*^2^(3) = 24.617, *p* < 0.001. It was also more difficult for *recently immigrated* parents than parents with an *immigrant-background* (*z* = 2.088, *p* = 0.004). The use of school apps or social media was the major facilitator for getting into contact with other parents (15.00% *refugee*, 20.00% *recently immigrated*, 34.78% *immigrant-background,* 9.52% *non-immigrant*). Major difficulties in reaching out to other parents included language barriers (40.00% *refugee*, 20.00% *recently immigrated*, 9.52% *non-immigrant*) as well as other parents not wanting to talk, discrimination, or a lack of socializing possibilities (25.00% *refugee*, 15.00% *recently immigrated*, 4.35% *immigrant-background*, 9.52% *non-immigrant*).

## 4 Discussion

To our knowledge, this is the first study providing quantitative information on RRI parents’ resources, academic engagement, and their relations to their children’s school adjustment in elementary schools in lower-income neighborhoods of a higher-income country. In our study, some of RRI and immigrant-background parents’ resources (German language skills, extent of attending local social support groups) were related to their academic engagement. However, parents’ academic engagement was unrelated to their children’s school adjustment. Between groups, we found several indicators that refugee parents may put a stronger emphasis on their children’s education. However, they also had fewer resources, were less engaged in school, more often considered schools to be primarily responsible for their children’s education, and most often wanted their children to pursue studies after school. Recently immigrated parents also reported fewer resources than immigrant-background parents and non-immigrant parents, but higher attendance of local social support groups than non-immigrant parents as an additional resource. They also saw more often their responsibilities in motivating their children and checking their homework. RRI parents also more often initiated contact with the class teacher and relied on information from direct contact than immigrant-background and non-immigrant parents. While it was easy for almost all parents to get in contact with the class teacher, it was more difficult for refugee parents to get in contact with other parents than for all other groups. Regardless of the study group, almost all parents in our study emphasized their willingness to be academically engaged. In many aspects, RRI parents also displayed similar resources as immigrant-background and non-immigrant parents. Parents’ most frequent stressors were time constraints and responsibility for their children or household chores.

### 4.1 Parents’ academic engagement and resources

#### 4.1.1 Home engagement

Even though all groups differed considerably regarding their immigration experiences, we found no group differences in their home engagement. This supports previous findings that RRI and immigrant-background parents exhibit high levels of home engagement (e.g., Baird, 2015; Schlaich, 2021; Vera et al., 2012). Contrary to findings that refugee and immigrant-background parents perceived themselves as not knowledgeable enough to assist their children with homework (Barglowski, 2019; Shamim et al., 2020), we found that high numbers of RRI and immigrant-background parents engaged in their children’s homework and considered this to be their task. This might be a general trend. An alternative explanation could be that, due to COVID-19, parents were encouraged to spend more time supervising their children’s homework. Noticeably, we found no difference in parents’ formal education between groups, which might have led to more homogeneous results. Knowledge of the neighborhood became evident in the places parents visited with their children. Refugee parents had taken their children to fewer places when compared to all other groups. Additionally, the extent of attending local social support groups predicted the extent to which the parents went on outings with their children. We found hints that going on more outings with their children might reduce their children’s hyperactivity/inattention. This supports the results of a recent meta-analysis, in which spending time in nature was repeatedly linked to reduced ADHD symptoms (Hood and Baumann, 2024). Future research should reassess this. Surprisingly, we also found that German language skills were negatively related to the extent parents went on outings with their children. These parents might have to fulfill concurrent tasks (e.g., translating for friends or relatives, working [multiple jobs]) and thus have less time to spend with their children. This would be in line with previous findings highlighting time constraints (Gandarilla Ocampo et al., 2021; Rah et al., 2009; Shamim et al., 2020). Future research should also look at parents’ specific tasks. Albeit insignificant, we found hints that the value the parents placed on their children’s grades and their knowledge of the education system may positively influence the extent to which they read with their children. In future research, those relations should be reassessed.

#### 4.1.2 School engagement

Following several previous studies (e.g., Cranston et al., 2021; Rah et al., 2009; Turney and Kao, 2009), we found that parents’ language skills positively predicted RRI and immigrant-background parents’ school engagement. Additionally, RRI parents more often initiated contact with the class teacher and relied on information from direct contact instead of written information than immigrant-background and non-immigrant parents. Both underscore that schools may foster parents’ school engagement by diminishing language barriers, which is in line with previous studies (Cranston et al., 2021; Rah et al., 2009). Also in line with previous literature, we found that refugee parents were the least engaged in school (Cureton, 2020; Koyama and Bakuza, 2017; Zengin and Akdemir, 2020) and most often considered the school to be responsible for their children’s learning (Snell, 2018). However, we do not know whether refugee parents were less engaged in school because they did not consider themselves responsible, or if other responsibilities hindered them from being engaged in school. For example, only 13.04% of all refugee parents had a permanent residency status, which is likely tied to many other worries and responsibilities. As suggested by previous studies (Baird, 2015; Vera et al., 2012), refugee parents might also favor home engagement over school engagement. In line with this, refugee parents in our study considered supporting their children’s learning and checking homework their most important tasks. However, almost all refugee parents fully agreed to the statement that they would like to be engaged in school, whereas only 17.65% mentioned time constraints. This suggests that schools may be successful in motivating refugee parents to be engaged in school, for example by directly inviting them (Walker et al., 2005). Additionally, the extent to which the parents attended local social support groups positively predicted their school engagement. Possibly, being part of a local community might increase RRI parents’ interest in becoming (more) engaged in school. Alternatively, parents who engage more in school might also be more engaged in the neighborhood. Compared to the other groups, it was more difficult for refugee parents to get in contact with other parents at school. This suggests that especially local social support groups could be a resource for refugee parents. Overall, our results suggest that especially parents’ German language skills and their local social support may be relevant to their academic engagement.

#### 4.1.3 No relations between parents’ academic involvement and children’s school adjustment

We found no relations between RRI and immigrant-background parents’ academic engagement and children’s school adjustment, which contrasts several studies (e.g., Areepattamannil and Lee, 2014; Jeynes, 2003; McBrien, 2005) but also is in line with few other studies (e.g., Hancorn, 2024). We have several possible explanations for these findings: First, it is likely that parents socialized in non-Western cultures are differently engaged in their children’s education than measured with items posed in questionnaires that refer to Western school systems, as has already been found in previous studies (Cureton, 2020; Gandarilla Ocampo et al., 2021; Schlaich, 2021). Such engagement strategies may include emotional and moral support, role modeling, conversations, encouragement, or decision-making (Malaeb and Ware, 2023). The idea of being engaged via emotional and background support is in line with the parents’ replies to our open-ended questions (e.g., parents most often replied that it was their duty to support their children). Future research on RRI parents’ academic engagement could put even more emphasis on the cultural differences in parents’ academic engagement. Second, RRI and immigrant-background children’s school integration, especially in the above-mentioned neighborhoods, may be fostered by factors other than parents’ academic engagement. Especially in elementary schools, children may be influenced by factors rooted in the school environment and teachers, such as teacher support, classroom climate, or peer relationships (e.g., Ialuna et al., 2024). Such factors might compensate for a lack of certain parent engagement strategies. Third, especially teachers working in schools located in lower-income and multiethnic neighborhoods are familiar with working with children from families with diverse immigrant backgrounds. The teachers might have found distinct ways to foster the children’s school adjustment without their parents’ academic engagement. Fourth, in schools with many lower-performing students, it may be easier for students to achieve good grades than in schools with many higher-performing students. Due to the unique school environments, children may be perceived as better-performing and better adjusted than they would be perceived within school environments with many high-performing children. Previous research has already linked teachers’ perception of student behaviors to environmental factors (e.g., Pas and Bradshaw, 2014). Moreover, teachers may adjust curricula to the unique composition of RRI children in these schools and may support them differently than teachers in schools with a high composition of non-immigrant children would do. Indeed, teachers were found to have lower academic demands on refugee children and rather focus on their well-being, leading to better socio-emotional adjustment of these students (Šeďová et al., 2025). Future research should also address such relevant factors and examine possible moderating or mediating effects of community and school environments.

#### 4.1.4 Parents’ resources

Both refugee and recently immigrated parents reported fewer resources (German language skills, local social support) than immigrant-background and non-immigrant parents, but did not differ from each other. This supports evidence by previous studies (e.g., Baker et al., 2021; Cranston et al., 2021; Rah et al., 2009; Shamim et al., 2020; Ziaian et al., 2018) and suggests that certain resources may not be flight-related but relevant for RRI families in general. This leads to the question of whether support programs for refugees should be extended to all RRI families. Still, recently immigrated parents appeared to be better integrated into local support structures (i.e., local social support groups). They also reported a better knowledge of the education system than refugee parents. This supports the idea that exposure to flight-related risk factors (Bronstein and Montgomery, 2011) may relate to parents’ lower levels of parent- and school-related resources. Likely, better integrating refugee families into local support structures and providing more information on the local education system could be beneficial for their integration. Future research should also reassess different aspects of knowledge of the education system, as we measured this construct rather broadly. Surprisingly, we found no relations between most parent-related resources and their academic engagement, which contrasts with another recent study on refugee parents (Baker et al., 2021). However, Baker and colleagues collected data in 2016, when refugee parents might not have had sufficient time to settle. Contrary to previous studies (Stoessel et al., 2011; Vera et al., 2012), we also found no relations between parents’ formal education and their academic engagement as well as no group differences in parents’ formal education. Noticeably, most of these studies were conducted in the U. S., where having a lower income is tied to worse living conditions than in Germany (e.g., less access to the welfare system or health care). Thus, resources such as formal education, mental health, and social support of a person nearby might be more important for parents’ academic engagement in countries with health, immigration, and social policies similar to those of the U.S. than it is in welfare states such as Germany. In contrast to expectations that refugee parents’ most important stressors might be flight-related such as having experienced traumatic events or an uncertain residency status (e.g., Baker et al., 2021; Bronstein and Montgomery, 2011), RRI parents’ most frequent stressors were time constraints and responsibility for their children or household chores. This hints at a somewhat progressed level of integration. Additionally, attending local social support groups likely was an important resource for RRI parents in our study, as it positively related to both their home and school engagement.

In line with several other studies (e.g., Cureton, 2020; Ziaian et al., 2018), we found that refugee parents put a high emphasis on their children’s education (i.e., they most often wanted their children to obtain a university or college degree). Presumably, parents having resided in lower-income neighborhoods for a longer time or with more knowledge of the education system may be less optimistic about their children’s school trajectories. In line with previous literature (Shamim et al., 2020), refugee parents reported a lower knowledge of the education system. They also reported a lower school engagement and feeling less responsible for their children’s education while reporting higher emotional symptoms in their children. Parents who know little about the receiving country’s education system but have high ambitions for their children might place high expectations on them. Given these circumstances, these parents should ensure that they do not put too much pressure on their children.

Furthermore, most of RRI and immigrant-background parents in our study were raised in collectivist cultures. Our results hint at RRI parents relying to a higher degree on community resources to navigate educational challenges such as participation in local social support groups or more frequent and/or personal communication with teachers and other parents (e.g., Ressler, 2020). Our results further raise the question to which extent community resources may be as important to RRI and immigrant-background parents’ academic engagement as parent-related resources. Future research should analyze how community factors may influence RRI and immigrant-background parents’ academic engagement and resources.

#### 4.1.5 Signs of a progressed level of integration

Several indicators suggest a progressed level of integration of RRI parents in our study. Many Arabic-speaking refugee families came to Germany between 2014 and 2016. Since then, most families may have managed to settle. Consequently, many children had already participated in daycare and had been enrolled in a German school since first grade. As a resource, nearly all parents had someone to share their joy or worries with. Refugee parents’ most frequent stressors were time constraints and responsibilities instead of flight-related stressors. Almost all parents reported a high willingness to be engaged in school, which emphasizes their high motivation to support their children’s educational trajectories, despite facing many barriers and additional risks. This contrasts with the assumption still prevailing in German society that lower-income parents have fewer ambitions for their children or are less willing to support them (Civitillo and Jugert, 2022).

### 4.2 Children’s school adjustment

In contrast to most literature (e.g., Jeynes, 2003; McBrien, 2005), we found that RRI and immigrant-background parents’ academic engagement did not predict children’s school adjustment. There might be several (interactions of) influencing factors not accounted for in this study or individual to each family, resulting in no consistent patterns of linkages. More precisely, providing emotional support and intentionally spending time with the child, but less so the exact engagement strategy, might be important for the children’s academic development. Instead, literature hints at parents’ academic engagement being related to their children’s academic performance (e.g., Altschul, 2011; Jeynes, 2003). Lower school engagement did not seem to pose a risk to their children’s school adjustment in our study (see Altschul, 2011). Future research should reiterate this in samples that represent current proportions of refugee and immigrant families in lower-income neighborhoods of higher-income countries. Additional research is needed to support or undermine this idea. Compared to the other parents, refugee parents more often initiated contact with the class teachers or received information directly from them, which could both function as protective factors to their children’s academic development and hint at different communication patterns (Rah et al., 2009). We also found no group differences in children’s grades. This has two implications. First, refugee and immigrant children in lower-income neighborhoods might integrate better over time as suggested by previous literature (Lau et al., 2018). Refugee families in our study had already been residing in Germany for an average period of 6.35 years. Their children might have already adjusted to the German school system, their new surroundings, and the new language (e.g., Lau et al., 2018). Second, in these multiethnic environments, lessons might be adapted to diverse class compositions, which could lead to fewer differences in grades between RRI, immigrant-background, and non-immigrant children. In terms of socio-emotional adjustment, however, RRI parents reported more emotional symptoms in their children than non-immigrant parents, which is in line with previous literature (e.g., Chwastek et al., 2022) and, nonetheless, hints at additional immigration- and flight-related risk factors to the children’s mental health (e.g., Bronstein and Montgomery, 2011). Moreover, the correlations suggest that parents’ knowledge of school lessons’ content might be related to children‘s school adjustment (i.e., emotional symptoms, well-being at school, and grades). Further research may investigate this.

### 4.3 Limitations

Our study is cross-sectionally designed and thus does not allow for causal conclusions. Moreover, the generalizability of our results might be limited to RRI parents in lower-income neighborhoods of European higher-income countries. Importantly, RRI parents are a very heterogeneous group. Albeit we could show that many RRI parents struggle with similar issues, they may still show very distinct patterns of exposure to resources and risk factors. Furthermore, the results of RQ1 and RQ2 do not allow for specific conclusions on RRI parents, as regressions were conducted with all RRI and immigrant-background parents. Some of our items, which were based on our overall literature review, did not sum up into scales (including two of the SDQ subscales), as McDonald’s *ω*s were too low. However, it was necessary to incorporate adapted items into our study instead of only applying existing constructs, as RRI parents’ resources and needs may differ depending on their living environments, their time of arrival in the receiving country, and support currently provided by the government. This underlines the importance of flexible and thorough research approaches such as taking alternative parental engagement strategies into account. We addressed this by adding open-ended questions. Additionally, several refugee parents replied very briefly to these open-ended questions. Future research may profit from giving parents more concise explanations of what they are asked to describe. Moreover, statistical evidence suggests that women and younger parents are more likely to be engaged in their children’s education than men and older parents (Chipalo, 2024). We reduced bias by examining academic engagement within the whole family as well as examining key resources for both the interviewee and the partner (i.e., language skills, formal education). We further did not account for age differences, as we already divided groups by time spent in Germany and because our study has an exploratory approach, with a focus on parents’ academic engagement and resource variables. However, future research should examine gender and age differences. Results may help identify suitable supportive measures for respective groups of RRI parents. Notably, more quantitative studies with better culturally adapted items are needed to obtain more accurate and reliable quantitative measurements of academic engagement for RRI parents.

## 5 Conclusion

With a focus on RRI and immigrant-background families in lower-income neighborhoods, our study examines the relations between parents’ academic engagement, their resources, and how both relate to their children’s elementary school adjustment. Despite facing significant resource constraints, our findings suggest that RRI parents demonstrate a willingness to be actively engaged in their children’s education. However, the refugee parents in our study also adopted a somewhat different view on their role in their children’s education when compared to immigrant-background and non-immigrant families. We found no link between children’s school adjustment and their parents’ academic engagement. Future research should further investigate the potential pathways of how RRI parents’ academic engagement may relate to their children’s school adjustment, especially with regard to better culturally adapted forms of engagement. Our results further raise the question of to what extent (a) community resources contribute to the academic engagement of RRI and immigrant-background parents and (b) school factors may buffer (a lack of) parents’ academic engagement, especially in elementary schools with high proportions of RRI children.

In sum, our study contributes to the development of approaches toward a successful school adjustment of RRI and immigrant-background children in lower-income neighborhoods of higher-income countries. Our findings suggest that measures aimed at supporting RRI families may consider alternative, better culturally adapted, forms of parents’ academic engagement and focus on strengthening parents’ resources such as the receiving country’s language proficiency, community support, and information transfer. Our findings indicate that (a) increasing RRI and immigrant-background parents’ availability of resources could facilitate their academic engagement, (b) RRI and immigrant-background children may adjust to school independently of their parent academic engagement as measured, (c) that the underlying mechanisms facilitating children’s school adjustment and the potential contribution of unmeasured (e.g., better culturally adapted) engagement forms warrant further investigation, and (d) that school- and community-level factors may compensate for limited parental resources.

## Data Availability

The datasets presented in this article are not readily available because the participants of this study did not give written consent for their data to be shared publicly. Due to the sensitive nature of the research supporting data is not available. Requests to access the datasets should be directed to SC, sandy.chwastek@ruhr-uni-bochum.de.
